# Distribution of stress on TMJ disc induced by use of chincup therapy: assessment by the finite element method

**DOI:** 10.1590/2177-6709.22.5.083-089.oar

**Published:** 2017

**Authors:** Flávio Siqueira Calçada, Antônio Sérgio Guimarães, Marcelo Lucchesi Teixeira, Flávio Atsushi Takamatsu

**Affiliations:** 1 Associação Paulista de Cirurgiões-Dentistas, Curso de Especialização em Ortodontia (São José dos Campos/SP, Brazil).; 2 Faculdade de Medicina e Odontologia São Leopoldo Mandic, Programa de Mestrado em Disfunção Temporomandibular e Dor Orofacial (Campinas/ SP, Brazil).; 3 Faculdade de Medicina e Odontologia São Leopoldo Mandic, Disciplinas de Oclusão e Clínica Protética (Campinas/ SP, Brazil).; 4 Faculdade de Medicina e Odontologia São Leopoldo Mandic, Centro de Pesquisas Odontológicas (Campinas/ SP, Brazil).

**Keywords:** Finite element analysis, Temporomandibular joint disc, Mechanical stress

## Abstract

**Objective:**

To assess the distribution of stress produced on TMJ disc by chincup therapy, by means of the finite element method.

**Methods:**

a simplified three-dimensional TMJ disc model was developed by using Rhinoceros 3D software, and exported to ANSYS software. A 4.9N load was applied on the inferior surface of the model at inclinations of 30, 40, and 50 degrees to the mandibular plane (GoMe). ANSYS was used to analyze stress distribution on the TMJ disc for the different angulations, by means of finite element method.

**Results:**

The results showed that the tensile and compressive stresses concentrations were higher on the inferior surface of the model. More presence of tensile stress was found in the middle-anterior region of the model and its location was not altered in the three directions of load application. There was more presence of compressive stress in the middle and mid-posterior regions, but when a 50^o^ inclined load was applied, concentration in the middle region was prevalent. Tensile and compressive stresses intensities progressively diminished as the load was more vertically applied.

**Conclusions:**

stress induced by the chincup therapy is mainly located on the inferior surface of the model. Loads at greater angles to the mandibular plane produced distribution of stresses with lower intensity and a concentration of compressive stresses in the middle region. The simplified three-dimensional model proved useful for assessing the distribution of stresses on the TMJ disc induced by the chincup therapy.

## RESUMO

**Objetivo:**

avaliar, por meio do método dos elementos finitos, a distribuição das tensões no disco articular produzidas pela mentoneira ortopédica.

**Métodos:**

um modelo tridimensional simplificado do disco articular foi desenvolvido com o *software* Rhinoceros 3D e exportado para o *software* ANSYS. Uma carga de 4,9 N (500 gf) foi aplicada na superfície inferior do modelo, com inclinação de 30, 40 e 50^o^ em relação ao plano mandibular Gônio-Mentoniano (GoMe). O ANSYS analisou, por meio do método dos elementos finitos, a distribuição das tensões presentes no modelo do disco articular para as diferentes angulações.

**Resultados:**

os resultados mostraram que a concentração das tensões de tração e compressão foi maior na superfície inferior do modelo. A tensão de tração foi mais presente na região média-anterior do modelo, e sua localização não se alterou nas três direções da aplicação da carga. A tensão de compressão foi mais presente nas regiões média e média-posterior do modelo, mas quando a carga a 50^o^ foi aplicada, ela se concentrou na região média. As intensidades das tensões de tração e compressão diminuíram progressivamente à medida que a carga foi aplicada mais verticalmente.

**Conclusão:**

as tensões induzidas pela mentoneira ortopédica se localizaram principalmente na superfície inferior do modelo. As cargas com maior angulação em relação ao plano mandibular produziram uma distribuição de tensões com menor intensidade e uma concentração da tensão de compressão na região média do modelo. Um modelo tridimensional simplificado se mostrou útil na avaliação da distribuição das tensões no disco articular induzidas pela mentoneira ortopédica.

## INTRODUCTION

The prevalence of Class III malocclusion varies, according to the literature, from 1 to 5% for populations of Caucasian descent,^1^ and from 2 to 13% for Asiatic populations.^2^ Skeletal Class III malocclusions occur as a result of: deficient maxillary growth; exacerbated mandibular growth or an association of both.^3^ Treatment for mandibular prognathism involves orthopedic interventions, such as use of the chincup therapy^4^ or reverse traction of the maxilla.^5^

The chincup therapy has been described in the literature since the beginning of the 19th Century, as a clinical procedure to control mandibular growth in young patients with mandibular prognathism.^6^ The chincup therapy effects on craniofacial growth and temporomandibular joint (TMJ) have been thoroughly investigated by cephalometric analyses and experiments in animals.^7^ ^,^ ^8^ Results such as morphologic alterations and mandibular growth inhibition have been described in the literature.^9^ ^,^ ^10^ The orthopedic effects of the appliance essentially depend on the magnitude and direction of the forces applied, and this fact in some ways may explain the diversity of results found with the use of the chincup therapy in Class III patients.^3^ ^,^ ^4^ ^,^ ^7^ Bone remodeling is directly related to the induction of stresses in the skeletal structures,^11^ therefore, to gain knowledge, the distribution of stresses imposed by the chincup therapy should be investigated, thus promoting better understanding of the mandibular bone remodeling mechanism and providing clinicians with guidance on therapy with this appliance.^12^

**Figure 1:**
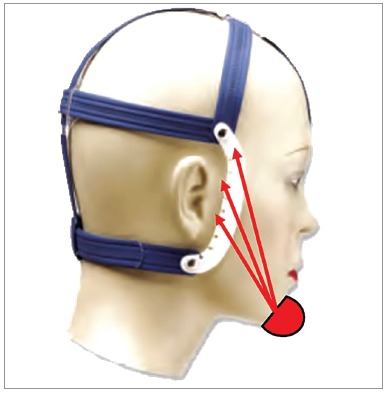
Chincup therapy with Interlandi’s cap in different directions of load application.

The use of the chincup therapy in the orthodontic clinic on several occasions is questioned about the possibility of being a risk factor for temporomandibular disorders (TMD), because this device adds extra loads to the TMJ.^13^ One of the functions of the TMJ disc is to absorb the stress imposed on the joint and dissipate it to the surrounding structures.^14^ Thus, to know the intensity and distribution of these stresses in the TMJ disc is fundamental to understand how this structure behaves during the use of chincup therapy. Therefore, this study was conducted aiming to use finite element method (FEM) to evaluate the stresses imposed on the TMJ disc by the use of chincup therapy, with load application in different directions (Fig 1).

## MATERIAL AND METHODS

**Figure 2:**
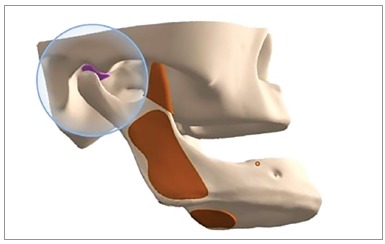
CAD image, with the articular disc model highlighted.

A computed three-dimensional model was constructed with the purpose of analyzing the stresses distribution on the TMJ disc induced by the use of chincup therapy. Computed tomography images of a dry, young skull provided the data required for constructing the mandible and glenoid fossa. The TMJ disc was graphically designed in the space between the condyle and glenoid fossa, with the aid of a CAD (computer-aided design) software (Rhinoceros 3D, Robert McNeel & Associates, Seattle, USA). Its biconcave morphology was based on figures taken from an anatomy atlas^15^ and its mean thickness was 2 mm^16^ (Fig 2).

**Figure 3:**
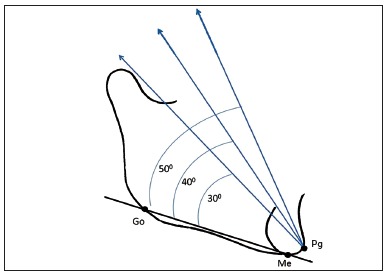
Load applied in Pg, at angles of 30, 40 and 50 degrees to the mandibular plane (GoMe).

**Figure 4:**
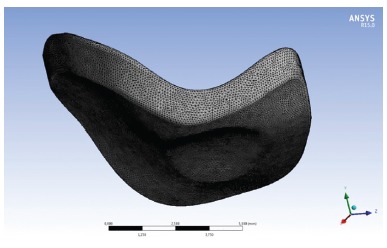
Discretized TMJ disc model.

The model was exported from Rhinoceros to ANSYS software (Swanson Analysis Systems Inc., Houston, USA), which performed the simulations and by means of FEM, calculated the stresses present on the TMJ disc model. A 4.9N (500gf) load was applied on the cephalometric point Pogonion (Pg), at angulations of 30, 40 and 50 degrees to the mandibular plane (GoMe) of the computed model (Fig. 3). Decomposition of the load on the *y* and *z* axes of the ANSYS software was equal to: 3.092N y / 3.801N z; 3.705N y / 3.206N z and 4.206N y / 2.514N z, for the 30, 40 and 50 degree angles respectively. After obtaining the vectors of the load on TMJ disc on the left side, the remainder of the model was isolated and discretized, producing a mesh with 1,256,630 elements and 1,737,367 nodes (Fig 4). Movement was restricted on the superior surface of the TMJ disc model, to prevent its displacement during the simulations. The loads were applied directly on the inferior surface of the TMJ disc model, specifically in the area that remains in contact with the superior surface of the condyle, according to the vectors previously obtained. This was done with the purpose of testing the possibility of a simplified three dimensional model satisfactorily responding to the computed simulation researches.

The mechanical properties of the TMJ disc were defined as Young’s modulus (E) of 40 MPa, and Poisson’ ration (y) of 0.4^17^. The model was assumed to have isotropic properties and linear elastic deformation^17^. The stresses distribution evaluated by FEM, contemplated the maximum principal stress (tensile stress) and the minimum principal stress (compressive stress) on the superior and inferior surfaces of the TMJ disc model.

## RESULTS

**Figure 5:**
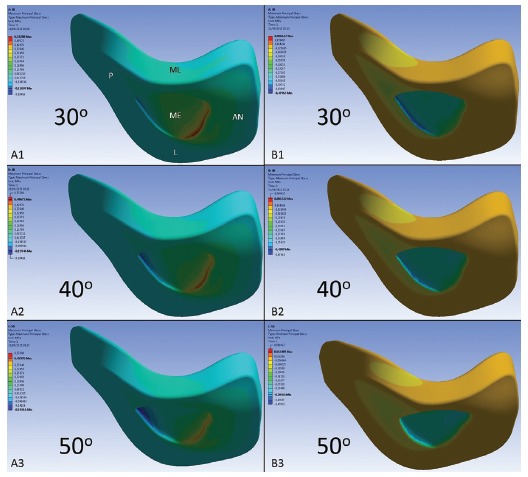
Inferior surface of the three-dimensional TMJ disc model, showing distribution of the stresses. A1 and B1; A2 and B2, and A3 and B3 tensile and compressive stresses respectively for loads applied at 30, 40 and 50-degree angles to the mandibular plane (GoMe). In A1: P, L, ME, ML and AN represent the posterior, lateral, middle, medial and anterior regions of the model.

In Figure 5, A1, A2 and A3 shows the tensile stress distribution on the inferior surface of the three-dimensional TMJ disc model when the loads were applied in the directions of 30, 40 and 50 degrees to the mandibular plane (GoMe). Basically, the tensile stresses were present in the mid-anterior region of the model, and there was no change in the position of their location when the direction of the loads varied. The most intense tensile stress, reaching 0.532 MPa, was observed when a load was applied at an angle of 30 degrees. For the 40 and 50-degree simulations, the intensity of these stresses progressively diminished, attaining 0.496 and 0.459 MPa, respectively (Fig 7).

**Figure 6:**
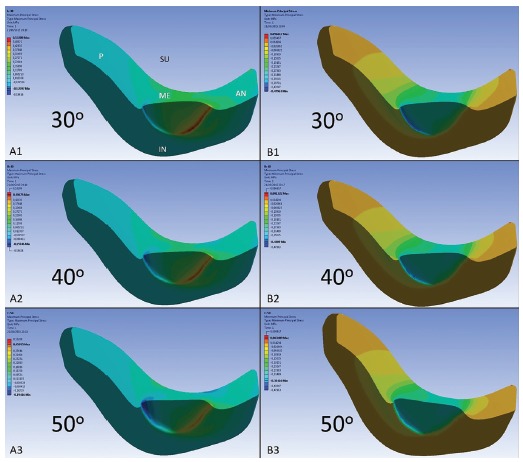
Longitudinal cut of the three-dimensional TMJ disc model. A1 and B1; A2 and B2, and A3 and B3 tensile and compressive stresses respectively for loads applied at 30, 40 and 50-degree angles to the mandibular plane (GoMe). In A1: P, AN, ME, SU and IN represent the posterior, anterior, middle, superior and inferior surfaces of the model.

**Figure 7:**
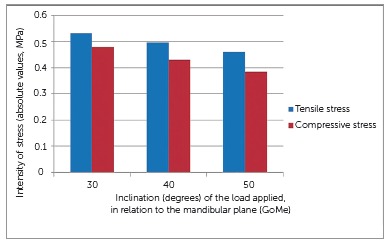
The effect of the inclination of the load applied to the three-dimensional TMJ disc model.

In Figure 5, B1, B2 and B3 shows the compressive stresses on the inferior surface of the three-dimensional TMJ disc model in the different directions of load application. These stresses were basically located in the middle and mid-posterior regions of the inferior surface of the model. When the load was applied at an angle of 30^o^, more intense compressive stress was shown on the model, attaining - 0,479 MPa. As the load was applied at 40 and 50-degree angles, the intensity of compressive stresses progressively diminished, attaining - 0.430 and - 0.384 MPa, respectively (Fig 7). When the direction of simulation was more vertical to the mandibular plane (50^o^), the compressive stresses were mainly located in the middle region of the model.

Figure 6 illustrates a longitudinal cut of the computed TMJ disc model, showing that the tensile (A1, A2 and A3), and compressive (B1, B2 and B3) stresses were more often present on the inferior surface of the model, when compared with the superior surface, for all the simulations performed.

## DISCUSSION

Computed simulations analyzed by means of the FEM have been successfully used in the area of health care over the last two decades,^18^ and the publication of validation studies has contributed to the scientific community accepting the importance of the results found with this technique.^19^ ^,^ ^20^ The possibility of testing hypotheses and changing variables in computed models allows for conducting studies that would never be possible to conduct in living beings.^21^ ^,^ ^22^

The morphological changes and inhibition of mandibular growth due to the use of the orthopedic chincup force have been demonstrated in various clinical studies, with the use of cephalometry.^7^ ^,^ ^9^ ^,^ ^10^ These changes are greater and more frequent in the condyle, mandibular ramus and body, than in the glenoid fossa, and at the cranial base.^23^ For the bone remodeling process to occur, both tensile and compressive stresses need to be present in the affected area.^12^ The results found in the present study confirmed this hypothesis, since they showed that these stresses were more often present on the inferior surface of the TMJ disc model, which remains in contact with the condyle, in all the performed simulations, and did not change with the shift in direction of the tested loads.^13^ Therefore, as in normal function,^24^ ^-^ ^27^ the TMJ disc absorbs the load and distributes the stresses produced by chincup therapy, by transferring lower intensity stresses to glenoid fossa and cranial base.

The possibility of chincup therapy causing or contributing in some way to the appearance of signs or symptoms of TMD is a frequent questioning of clinicians, due to the addition of extra load in the TMJ. When analyzing studies using FEM in functional movements such as mandibular opening and closing of a masticatory cycle, the stresses travel through the different regions of the TMJ disc, but basically the middle region of this structure presents compressive stresses,^28^ and the anterior region, tensile stresses - the latter due to contraction of the lateral pterygoid muscle.^26^ In the present study, the tensile stresses were present in the middle-anterior region for all the simulations performed; and the compressive stresses were located in the middle and mid-posterior regions for the simulations at 30 and 40 degrees. When a load was applied at an angle of 50 degrees, this stresses was basically concentrated in the middle region of the TMJ disc model. Therefore, the indication of the chincup therapy in mandibular prognathism in patients with vertical growth, which require a more vertical load direction of the device, will present a distribution of stresses in the TMJ disc similar to that found in chewing. While patients with horizontal growth, who will require loads with more horizontal directions, will present a higher concentration of the compression stresses in the mid-posterior region of the TMJ disc.

The possibility of chincup being a deleterious factor for TMJ should also be evaluated by the intensity of stresses added by chincup therapy. In this study the simulated load in the three-dimensional model was 4.9 N and produced the highest tensile and compression stresses in the TMJ disc, respectively 0.532 and - 0.479 MPa, when a load with 30^o^ of direction was applied. These stresses decreased in intensity as the load became more vertical (Fig 7). Studies using computerized simulation, which evaluated the chewing function, estimated loads up to 80 N, and found stresses in the three-dimensional models of the TMJ disc between 1 and -1 MPA.^28^ ^,^ ^29^ Thus, a properly shaped and well-positioned TMJ disc, as defined in our study, is capable of absorbing the load and distributing the stresses produced by the chincup therapy, because it is less intense than produced during clewing, even when a more horizontal load of the appliance is indicated. Another positive factor is the time of use of the chincup therapy: its installation occurs in childhood, when the modulus of elasticity of the TMJ disc is smaller, therefore with a greater capacity to absorb loads.^27^

In the present study, the authors applied a load of 4,9N on one single TMJ disc model. This was done with the intention of evaluating whether a load with higher intensity could cause a different stresses distribution to that found in similar studies, and whether a simplified three-dimensional model would satisfactorily meet the requirements of researches involving computed simulations. The values of the tensile and compressive stresses found in this study were higher when compared with similar published studies^15^- demonstrating that the greater the intensity of the appliance load, the greater was the intensity of the stresses present on the TMJ disc; however, the distribution of stresses in the different regions of the TMJ disc showed no significant difference. Therefore the direction of load application is the most important variable for achieving better balance in stress distribution on the TMJ disc during the chincup therapy; thus, it should not be disregarded during the treatment of mandibular prognathism. Finally, the simplified three-dimensional model used in this study, analyzed by FEM, proved to be a useful tool for studying and understanding the distribution of stresses present on the TMJ disc, and obtained similar results to those of other published studies.^13^ This finding suggests that researches with comparative computed simulations may use less complex models in their methodology.

In spite of the benefits a study of this type provides in recognizing the biomechanical effects on components of the TMJ, further researches should be conducted with the aim of perfecting the construction of three-dimensional models; discovering more precisely the mechanical properties of human tissues, and validating the results obtained with computed simulations.

## CONCLUSION

After analyzing the results obtained, it can be concluded that:

a) The inferior surface of the TMJ disc model presented higher concentration of stresses than the superior surface, when it was submitted to the simulations.
b) The middle-anterior region of the TMJ disc model presented tensile stresses in all the simulations, and its position did not change in the different directions of load application.
c) The middle and mid-posterior regions of the TMJ disc model presented compressive stresses; however, when the simulated load became more vertical to the mandibular plane, these stresses tended to concentrate in the middle region.
d) The intensity of the compressive and tensile stresses present on the TMJ disc model diminished as the simulated loads became more vertical to the mandibular plane.

Thus, the chincup therapy with a more vertical traction on the mandibular plane (GoMe), for treatment of mandibular prognathism, promote on the TMJ disc lower intensity of the tensile and compression stresses.
